# Halfway to Automated Feeding of Chinese Hamster Ovary Cells

**DOI:** 10.3390/s23146618

**Published:** 2023-07-23

**Authors:** Simon Tomažič, Igor Škrjanc

**Affiliations:** Faculty of Electrical Engineering, University of Ljubljana, 1000 Ljubljana, Slovenia; igor.skrjanc@fe.uni-lj.si

**Keywords:** spectroscopy, Raman, modelling, soft sensor, variable selection, outliers, simulator, kinetic model

## Abstract

This paper presents a comprehensive study on the development of models and soft sensors required for the implementation of the automated bioreactor feeding of Chinese hamster ovary (CHO) cells using Raman spectroscopy and chemometric methods. This study integrates various methods, such as partial least squares regression and variable importance in projection and competitive adaptive reweighted sampling, and highlights their effectiveness in overcoming challenges such as high dimensionality, multicollinearity and outlier detection in Raman spectra. This paper emphasizes the importance of data preprocessing and the relationship between independent and dependent variables in model construction. It also describes the development of a simulation environment whose core is a model of CHO cell kinetics. The latter allows the development of advanced control algorithms for nutrient dosing and the observation of the effects of different parameters on the growth and productivity of CHO cells. All developed models were validated and demonstrated to have a high robustness and predictive accuracy, which were reflected in a 40% reduction in the root mean square error compared to established methods. The results of this study provide valuable insights into the practical application of these methods in the field of monitoring and automated cell feeding and make an important contribution to the further development of process analytical technology in the bioprocess industry.

## 1. Introduction

Chemometrics, which deals with the application of various mathematical and statistical methods, could be described by a broad definition in which the most important part is the application of a multivariate data analysis to data relevant to chemistry [1]. The multivariate statistical data analysis is a powerful tool for analysing and structuring data sets obtained from different measurement systems and for building empirical mathematical models that can predict, for example, the values of important properties that cannot be measured directly [2,3]. Multivariate calibration is often used in the industry for the rapid online determination of important process parameters and critical quality characteristics and enables non-destructive measurements, online monitoring and process control.

In analytical chemistry, molecular spectroscopic methods, including infrared, near-infrared and Raman spectroscopy, are widely used to determine the molecular structure of various substances [4,5,6]. These methods work by assessing the radiant energy that is either absorbed or scattered when excited by a high intensity monochromatic beam that induces a transient energy state in the molecule. The process of Raman scattering occurs when the material under investigation is exposed to monochromatic light, causing a tiny percentage of the light to be inelastically scattered at wavelengths other than the incident light.

Raman spectroscopy is an optical method that enables the non-destructive investigation of molecular structures and chemical compositions. However, due to its low intensity, the study of Raman scattering requires the use of sophisticated instruments [7]. The data obtained from spectroscopy contain thousands of wavenumbers (variables) and measurements (observations), which requires multivariate analysis to determine the relationship between these variables [8,9]. Modern Raman instruments usually use a laser as the illumination source because of its high-intensity monochromatic properties. The wavelength of this laser can range from the UV (λ=200 nm) to the near-infrared (λ=1064 nm), but for pharmaceutical or biological applications, near-infrared wavelengths (λ=785 or λ=830 nm) are usually preferred to minimise fluorescence interference.

In bioprocess literature, spectroscopic sensors are sometimes referred to as soft sensors [10] because the spectroscopic data are modelled in software programmes that provide information analogous to that of hardware sensors. It is critical that data analysis models are used to extract the optimal amount of information from Raman spectra, an area that has received much attention in research [11]. The complexity and difficulties associated with interpreting results from Raman and IR spectroscopy can be mitigated by applying various data mining methods required for a more comprehensive understanding. These methods must be able to manage large multidimensional data sets while exploring the totality of spectral information [12].

Chemometric techniques, including the commonly used Partial Least Squares (PLS) [13,14] method, exploit the transformation capabilities of the principal component analysis (PCA). In this technique, the attributes of a data set are transformed into uncorrelated principal components, which allows a reduction in data dimensions with minimal loss of information. PCA-based techniques complemented by machine learning methods such as decision trees [15], Support Vector Machine (SVM) [16] and artificial neural networks (ANN) [17,18] allow for an even finer analysis. Additional preprocessing steps can be implemented, including normalisation and smoothing via *k*-th order Savitzky–Golay derivative [19], while model accuracy can be assessed by the standard error of calibration, factors used and coefficient of determination $\left(R^{2}\right)$.

The inherent complexity of spectral data derived from vibrational spectroscopic techniques, including IR, NIR and Raman, has sparked debates on the topic of variable selection in PLS regression models [20,21]. This complexity arises from the interference caused by the scattering of diffuse light, instrumental noise and overlapping absorption bands. Given this complexity, variable selection strategies focus either on single wavelengths (e.g., variable importance in projection [22]) or on informative spectral intervals (such as interval PLS [23]). These methods help to eliminate superfluous information, a concept introduced by Spiegelman et al. [24]. More recently, the technique of the Competitive Adaptive Reweighted Sampling (CARS) has proven its effectiveness in processing NIR and RAMAN spectra [25,26].

Certain Raman spectra obtained from the same sample may differ from the group due to factors such as instrumental artefacts and variations in the sample. These spectra are often referred to as unwanted spectra or outliers. Omitting these spectra is considered crucial before applying multivariate techniques to obtain the desired results.

Raman spectroscopy, known for its precise spectral features that correlate with the molecular structure of a sample, has demonstrated its strengths in a non-destructive analysis and its ability to work with aqueous systems. These properties make it particularly suitable for the study of cell cultures and tissues [27]. It is widely used for the study of polysaccharides, amino acids, alcohols and metabolites and has secured its position as an important process analytical technology (PAT) in the bioprocess industry [18,28,29]. The ability of inline Raman spectroscopy to monitor and adjust critical parameters in real time ensures consistent drug production.

Although mammalian cell cultures are widely used in the pharmaceutical industry to produce biological products such as antibodies and growth factors, the full potential of advances in process monitoring and control has not yet been realised [10,27]. Conventional methods, often based on offline sampling and manual calculations, are still widely used. In particular, mammalian cells are mainly used for the production of protein therapeutics, which account for 60–70% of biopharmaceuticals. These processes usually involve the delivery of glucose to CHO cells [30,31,32,33].

By using non-invasive real-time measurements PAT in conjunction with closed-loop feedback control, feeding strategies can be optimised to improve yield [29,34,35]. Raman spectroscopy plays an important role in this, as it enables in-situ measurements and process control in real time. In situ Raman measurements, first presented by [36], allow the simultaneous measurement of total cell density (TCD), viable cell density (VCD) and concentrations of glucose, glutamate, lactate and ammonia. This method has proven successful in monitoring mammalian cell cultures in bioreactors. Several successful examples can be found in recent literature [18,34,36]. Subsequent studies have extended this application from developmental scales of 3 to 15 L [27,34] to clinical production scales of 2000 L [37], demonstrating the scaling potential of this approach.

This manuscript represents a significant advance in the field of bioprocess technology by providing a comprehensive PLS model construction procedure for Raman spectroscopy that incorporates data preprocessing and outlier removal, thereby improving the understanding and control of bioprocess behaviour. In addition, the development of a simulator that incorporates CHO cell kinetics is an important contribution to the field. It paves the way for the development of a model predictive control system for the automated feeding of CHO cells, revolutionising the way we approach the automation and control of bioprocesses.

The paper is organized as follows. Section 2 and Section 2.1 describe the process of data acquisition and introduce the process of spectra processing, which is the initial step of data analysis. Section 2.2 explains the development of the PLS models for soft sensor design and different methods for variable selection in spectroscopic multivariate calibration. This subsection also discusses the process of identifying and removing outlier spectra to improve the robustness and accuracy of the PLS model. Section 3 discusses the CHO cell kinetics model required to develop an advanced simulation environment. Section 4 presents the results of the model construction and simulator implementation. Section 5 and Section 6 provide the discussion and concluding remarks.

## 2. Materials and Methods

### 2.1. Spectra Processing

The extensive research began with the systematic compilation of measurements and data obtained from the cultivation of CHO cells in a stainless steel bioreactor. The local pharmaceutical company, which was in charge of designing the experiment, played an important role. Our task, on the other hand, was to analyse the collected data, create the necessary models and establish a suitable simulation environment, which is described in this paper.

The cultivation of the CHO cells took place in a bioreactor with a volume of 10 L. To collect measurements (Raman spectra), the probe of a Kaiser RamanRXN2 spectrometer was inserted into the bioreactor. The RamanRXN2 spectrometer is a sophisticated analytical device that uses laser light with a wavelength of 532 nm. The resulting Raman spectrum is collected over a period of at least 30 min, a measure that improves the signal-to-noise ratio. It is important to note that Raman scattering, which is essentially inelastic photon scattering, is a rather small fraction compared to its elastic counterpart.

For data storage, a desktop computer with a Windows operating system was used, which was directly connected to the Raman spectrometer. Four different experiments were performed to grow the cells in the bioreactor, with each batch lasting about two weeks. The bioreactor contained CHO-S cell lines. This cell line is a sub-line of the original CHO-K1, with adaptations for suspension culture. CHO-S cells are commonly used in the industrial production of therapeutic proteins.

To maintain the optimal environment of the bioreactor, the pH and temperature were strictly controlled and nutrient dosing (glucose and glutamine) was conducted manually on a daily basis using reference measurements. A Roche Cedex Bio Analyzer, known for its reliability and precision, was used to record these reference measurements daily. This allowed for the accurate monitoring of parameters such as glucose and glutamine concentration, viable cell count and others.

**Figure 1:**
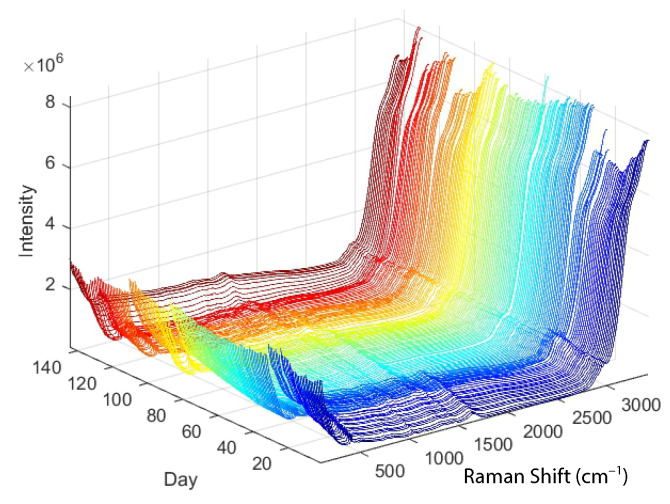
Spectra obtained with Raman spectroscopy (from four different batches where only spectra, which are used for training and validation, are shown).

The development of useful models depends on appropriate methods, but even more important is the selection of appropriate data. In our case, the raw data consist of the Raman spectra shown in Figure 1. For a first experiment, the choice between regression methods such as principal component regression, partial least squares or an artificial neural network may not be so important [27]. However, it is important that the selected independent variables (*x*-data) have a strong relationship with the dependent variables (*y*-data) to be modelled [38]. The choice of method then depends on the type and amount of data available.

In cases where the x-data for objects represent time series or digitised data from a continuous spectrum (e.g., Raman spectra, see Figure 1), possible pre-processing strategies could include smoothing or a transition to a first or second derivative. Smoothing attempts to reduce random noise by eliminating sharp peaks in the spectrum, while differencing brings relevant data to light despite noise amplification. The first derivative achieves alignment of spectra with different absorbance values that are shifted in parallel by cancelling out an additive baseline. A second derivative removes a constant and linear baseline. Each object vector, referred to as $x_{i}$, undergoes separate processes of smoothing and differentiation.

**Figure 2:**
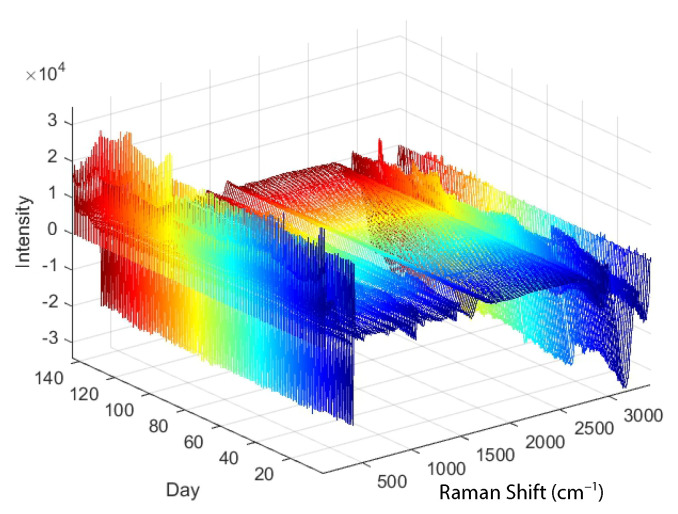
Raman spectra to which Savitzky–Golay filtering has been applied.

For both differentiation and smoothing, the Savitzky–Golay method is used. This is a method widely used in chemistry. This technique, a local polynomial regression using the method of least linear squares, requires x-values that are both exact and uniformly distributed. For each point, symbolised as *j* with value $x_{j}$, a linear combination is used to calculate the weighted sum of the neighbouring values. These weights determine whether smoothing or a derivative calculation is performed. Factors such as the number of neighbours and the polynomial order determine the strength of the smoothing. Choosing the right polynomial order is crucial, as incorrectly chosen higher order polynomials could misinterpret significant Raman bands as mere background. In the Savitzky–Golay method, a vector component $x_{j}$ is transformed by

(1) $$x_{j}^{*}=\frac{1}{N}\sum_{h=-k}^{k}c_{h}x_{j+h},\;$$

where $x_{j}^{*}$ is the new value (of a smoothed curve or a derivative), *N* is the normalisation constant, *k* is the number of neighbouring values (determining the size of the moving window) on each side of *j* and $c_{h}$ are the coefficients, which depend on the degree of the polynomial used and the objective (smoothing, first or second derivative). For example, if a second order polynomial is fitted through a window of five points (k=2), the following coefficients $c_{-2}$, $c_{-1}$, $c_{0}$, $c_{1}$, $c_{2}$ can be used for smoothing: −3, 12, 17, 12, −3, the first derivative: −2, −1, 0, 1, 2, and the second derivative: 2, −1, −2, −1, 2 [19]. Figure 2 shows the Raman spectra to which the Savitzky–Golay filtering was applied.

The process of pre-processing includes both filtering and normalisation, with the latter playing an important role. The reason for this is that even spectra recorded for the same material may demonstrate differences due to different recording times or unequal instrument conditions such as laser power and alignment. These variations can lead to different intensity values for spectra of the same material.

To compensate for these intensity differences, normalisation comes into play. This process ensures a maximum similarity of the intensity of a given Raman band of a given material when the spectra were taken under the same experimental parameters; however, some conditions are slightly different. Various normalisation methods are explored in the literature, including min-max normalisation, vector normalisation and Standard Normal Variate (SNV) normalisation. Of these methods, SNV normalisation is the most commonly used [39,40]. SNV normalisation works on the basis of the Equation (2), which can be outlined as follows:

(2) $${\hat{x}}_{j}^{*}=\frac{x_{j}^{*}-{\bar{x}}^{*}}{\sigma }\;\mathrm{where}\;\sigma =\sqrt{\frac{1}{N}\sum_{j=1}^{N}{\left(x_{j}^{*}-{\bar{x}}^{*}\right)}^{2}}\;\mathrm{and}\;j=1,2,...,N.\;$$

**Figure 3:**
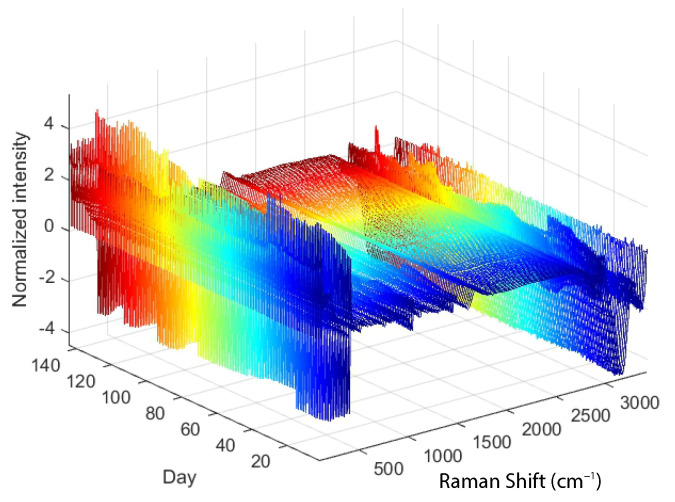
Raman spectra to which Savitzky–Golay filtering and SNV normalization are applied.

Figure 3 shows the Raman spectra for which SNV normalisation was performed in addition to Savitzky–Golay filtering.

### 2.2. Model Construction

The construction of predictive models for bioprocesses, particularly for the cultivation of CHO cells in bioreactors, has made significant progress through the application of chemometric methods to Raman spectroscopic data [38]. These models can predict several key variables such as the concentrations of glucose, glutamine, lactate and other biochemical parameters, as well as cell growth metrics such as total cell count (TCC) and viable cell count (VCC). Raman spectroscopy, a non-invasive, label-free technique, provides detailed chemical information about the bioprocess by recording the molecular vibrations of the components. The resulting Raman spectra serve as input data for the prediction model and provide a comprehensive, high-dimensional data set.

Model construction begins with a calibration phase in which known samples are analysed using Raman spectroscopy and appropriate laboratory tests. This process generates a set of reference data that includes Raman spectra and associated concentrations of glucose, glutamine, lactate and cell counts. Another way to collect reference measurements is to use a device such as Roche’s Cedex Analyzer. Once the reference data are prepared, multivariate analysis techniques such as Partial Least Squares Regression (PLSR) are used to build the predictive model. These methods work by identifying correlation patterns within the Raman spectra and relating them to the biochemical parameters of interest.

For more complex data sets or non-linear relationships, machine learning techniques such as Random Forest or SVM can be used. Advanced deep learning techniques such as Convolutional Neural Networks (CNN) are particularly effective for processing high-dimensional spectral data, as they can automatically extract meaningful features and improve prediction accuracy [18]. However, one must be aware that such a method of creating a model requires a large database, which is not always available.

This approach not only improves our understanding of the bioprocess, but also our control over it. The real-time predictive capability of the model leads to optimised and consistent bioproduction outcomes by enabling rapid, data-driven decision-making and process adjustments, thereby increasing bioprocess performance, reducing costs and improving product quality. The model is continuously refined as more data become available, improving its predictive power over time.

#### 2.2.1. Partial Least Squares

Partial Least Squares (PLS) is a statistical method that finds a linear regression model by projecting the predicted variables and the observable variables onto a new space. The method was first developed by Swedish statistician Herman Wold and has since been widely used in fields such as chemometrics, neuroimaging, bioinformatics and social sciences [41,42].

PLS simultaneously accounts for the covariance of both the independent variables (predictors) and the dependent variables (responses). This approach is advantageous when dealing with complex, multivariate data sets where the predictors are highly collinear or where there are more predictors than observations. The method can handle noisy and missing data, which makes it robust and flexible.

Partial Least Squares (PLS) regression is a multivariate technique that combines features of principal component analysis (PCA) and multiple linear regression. Although PCA is not explicitly used in the PLS method, the concept of extracting principal components or latent variables is central to both methods. In PCA, the goal is to find a small number of uncorrelated variables, called principal components, that explain most of the variation in the data. Each principal component is a linear combination of the original variables and is orthogonal to all other components. PLS works in a similar way, but instead of trying to explain as much of the variance in the predictor variables as possible, PLS tries to extract components that explain as much of the covariance between the predictor and response variables as possible. Essentially, PLS looks for directions in which the predictors not only explain a large part of their own variance (as in PCA), but are also highly correlated with the response. PLS regression can be summarised in the following steps:

- Standardisation of data: The first step in PLS regression is to standardise the predictor and response matrices. This ensures that the model is not overly influenced by variables that have large values or a large range of values.
- Extraction of PLS components: PLS decomposes the predictor and response matrices into a set of orthogonal components. These are linear combinations of the original variables that explain the maximum covariance between the predictors and the responses. The number of PLS components is chosen to optimise the predictive power of the model.
- Estimation of the PLS model: The PLS regression coefficients are estimated by relating the PLS components to the responses. These coefficients show the relationship between the changes in the predictor variables and the changes in the response variables.
- Prediction and validation: The PLS model can then be used to predict responses for new data. Cross-validation is often used to assess the predictive performance of the model and to determine the optimal number of PLS components.

In terms of its statistical properties, PLS is a form of regularised regression. Like other forms of regularisation, it can prevent overfitting by introducing some bias into the model, but it reduces the variance of the model and thus improves its predictive performance.

PLS has been extended to handle different types of data and different modelling scenarios. The most popular versions of PLS include PLS-DA (PLS Discriminant Analysis) [43] for classification problems and PLS-PM (PLS Path Modelling) [44] for structural equation modelling. These extensions have made PLS a versatile and powerful tool for multivariate analysis. When considering the use of PLS, it is important to understand its assumptions and limitations. Although PLS does not assume that predictors are independent or normally distributed, it does assume a linear relationship between predictors and responses. In addition, PLS may not work well with unrelated predictors because it attempts to use all predictors in the model, which can lead to overfitting. It is recommended to evaluate the performance of PLS against other multivariate methods such as principal component regression (PCR) or ridge regression to ensure that it is appropriate for a particular data set and research question.

The Nonlinear Iterative Partial Least Squares (NIPALS) algorithm is a common method for calculating PLS components. The goal is to find a set of components (also called latent vectors) that capture the covariance between the predictors and the responses. The algorithm of the simplified NIPALS method can be summarised in the following five points:

- Initialization:
  (3) $$X\in R^{n\times m},\;Y\in R^{n\times p},$$
  where *X* is a predictor matrix and *Y* is a response matrix.
- Selection of an initial column vector. Typically, the first column of the *Y* matrix represents the vector *u*:
  (4) u=Y[:,1]
- Iteratively compute the weights *w* and *t* until convergence:
  (5) $$w=\frac{X^{T}u}{u^{T}u}$$
  Normalize the weights:
  (6) $$w=\frac{w}{∥w∥}$$
  Compute the score vector:
  (7) t=Xw
  Reassign *u* as:
  (8) $$u=Y^{T}t/t^{T}t$$
  The iteration continues until the difference between the new and old score vectors falls below a certain threshold, indicating convergence.
- Deflate *X* and *Y*:
  Calculate the outer product of *t* and *p* (the loading vector for the *X*), then subtract it from *X*. Do the same for *Y* with *t* and *q* (the loading vector for the *Y*):
  (9) $$p=\frac{X^{T}t}{t^{T}t},\;q=\frac{Y^{T}t}{t^{T}t}$$
  (10) $$X=X-tp^{T},\;Y=Y-tq^{T}$$
  The iterations end when *X* (or *Y*) can no longer be deflated or when the number of extracted latent variables is enough to describe the data according to some criterion.
- Calculate the regression coefficients. Once all the latent vectors are extracted, the regression coefficients *B* can be calculated as:
  (11) $$B=W{\left(P^{T}W\right)}^{-1}Q^{T},$$
  where *W* is the matrix of weight vectors, *P* is the loading matrix of *X*.

**Figure 4:**
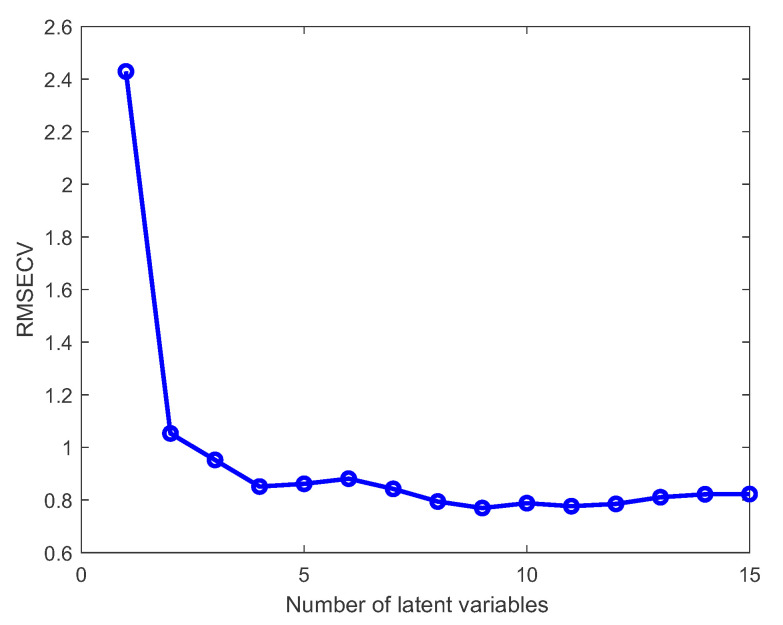
Finding the most appropriate number of latent variables in a PLS model.

The Root Mean Square Error of Cross-Validation (RMSECV), which is calculated during the creation of the PLS model, can be used as a criterion to find the right number of latent variables and prevent overfitting. For example, Figure 4 shows that in the case of a PLS model for glucose concentration, the most appropriate number of latent variables is four, as the RMSECV does not drop drastically after that.

#### 2.2.2. Selection of Key Variables

To further improve the PLS models and reduce the possibility of overfitting, the Variable Importance in Projection (VIP) and Competitive Adaptive Reweighted Sampling—Partial Least Squares (CARS-PLS) methods were used.

Variable Importance in the Projection is a popular method for assessing the importance of variables in a Partial Least Squares (PLS) regression model. PLS is a statistical approach used in predictive modelling where the prediction of a set of dependent variables from a set of independent variables is conducted through latent variable regression.

**Figure 5:**
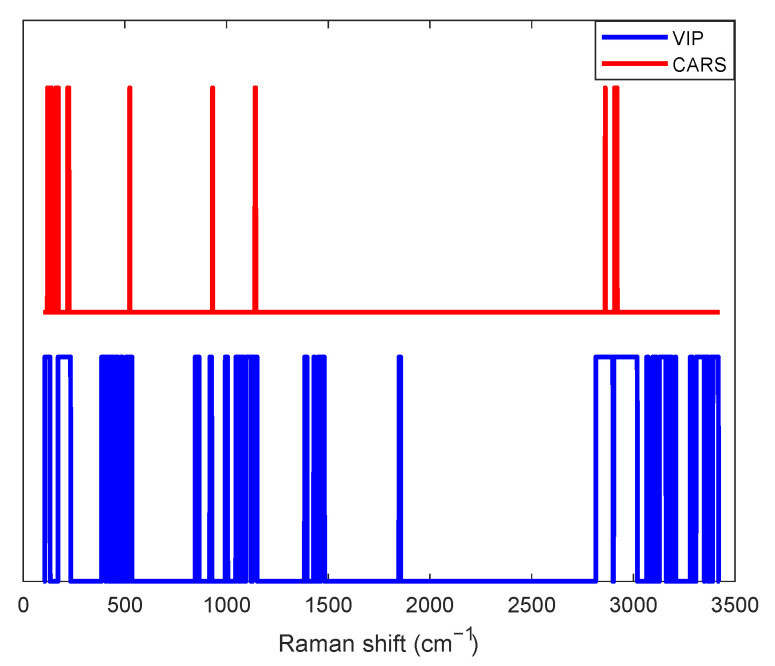
Key variables determined with the methods VIP and CARS for the PLS model of glucose concentration.

The VIP score for a variable is a measure of that variable’s contribution to the model, taking into account both its contribution to explaining the dependent variable and its contribution to explaining the independent variable. A high VIP score indicates that the variable is highly significant in the model (Figure 5 shows an example of selecting key variables in a PLS model of glucose concentration). However, the VIP method also has some disadvantages:

- Overemphasis on highly collinear variables: If variables are highly collinear, the VIP score can overestimate the importance of those variables and result in a model that may not be as accurate as possible. This can be problematic in areas where variables may be highly correlated, such as genomics or metabolomics.
- Unreliable with small data sets: The VIP method can be unreliable with small data sets because it depends on having enough data to estimate the PLS model accurately.

On the other hand, Competitive Adaptive Reweighted Sampling—Partial Least Squares is a more recent technique used for variable selection in spectroscopic multivariate calibration. It has gained considerable attention in the field of chemometrics. CARS-PLS was developed to overcome two major challenges in the analysis of spectroscopic data: high dimensionality and multicollinearity. These problems can lead to overfitting of the model, poor generalisation ability and difficulties in interpretation. The method CARS-PLS consists of two main stages:

- Competitive Adaptive Reweighted Sampling: This is a Monte Carlo-based sampling technique that helps identify relevant variables (wavelengths) for building the model. Initially, CARS assigns equal weights to all variables. Then, a set of subsets of variables is generated, each subset containing each variable with a probability proportional to its weight. A PLS model is created for each subset and its performance is evaluated. Based on the evaluation, the weights of the variables are updated—variables that frequently contribute to good models are given higher weights, while those that contribute to poor models are given lower weights. This process is repeated many times (usually thousands of iterations) until the best subset of variables is found.
- Partial Least Squares (PLS): After identifying the best subset of variables with CARS, a PLS model is built using only these selected variables (Figure 5). This model is simpler and less prone to overfitting than a model built with all variables. Moreover, because only relevant variables are included, the model is often easier to interpret.

The CARS-PLS method has been used successfully in many areas where spectroscopic data are used, such as pharmaceutical analysis, food quality control and environmental monitoring. However, like all methods, it has its limitations and assumptions. It assumes that there is a linear relationship between predictors and responses, and it may not work well if this assumption is not met. In addition, the performance of CARS-PLS may depend on the initial weights of the variables and the number of Monte Carlo iterations. Therefore, it is often advisable to make several runs of CARS-PLS with different initial settings and determine the consensus of the results.

Compared to VIP, CARS offers the following advantages:

- Better handling of collinearity: In contrast to the method VIP, CARS can better handle the problem of collinearity between variables.
- Simplicity and interpretability: CARS tends to lead to simpler and more interpretable models, which is of great importance in practical applications.
- Better performance on small data sets: CARS is not as reliant on large data sets as VIP and is therefore a more reliable method for variable selection on small data sets.
- More robustness: CARS is less prone to overfitting because it focuses on a subset of particularly relevant variables instead of considering all variables in the model.
- Adaptive: CARS is an adaptive method, able to adjust its selection as more data becomes available or the nature of the data changes.

#### 2.2.3. Removal of Outlier Spectra

**Figure 6:**
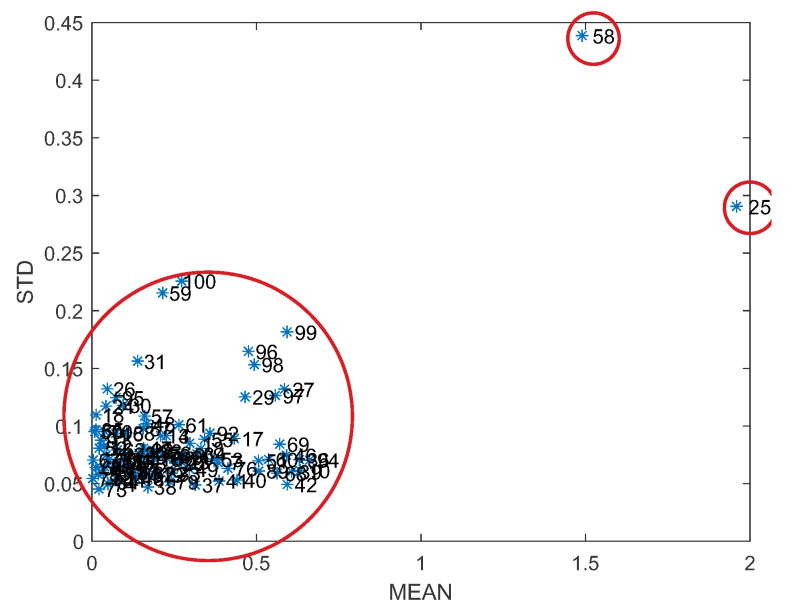
The mean error and standard deviation for all spectra.

The PLS model can be further improved by searching for spectra representing outliers. Therefore, a resampling method commonly used in statistics and machine learning was used, which can also be referred to as Monte Carlo cross-validation or repeated random sub-sampling validation. The outlier detection method consists of the following five steps:

- Partitioning: first, the original training dataset is randomly partitioned into a training dataset and a test dataset. For example, the partitioning could be 4:1, i.e., 80% of the data are used for training and 20% for testing. This partitioning is conducted many times, which is characteristic of a Monte Carlo approach.
- PLS modelling and prediction: A Partial Least Squares (PLS) regression model is built using the training data. This model is then used to make predictions for the test subset.
- Error calculation: The prediction errors for each spectrum in the test set are then calculated. Each spectrum will occur multiple times in different test sets; thus, an average error and standard deviation can be calculated for each spectrum across all iterations.
- Identification of outliers: Spectra that consistently produce high prediction errors (based on their average error or a combination of average error and standard deviation) can be considered outliers. These outliers represent spectra that are not well modelled by the PLS and thus affect the accuracy of the model. In Figure 6, for example, it can quickly be observed that the 25th and 58th spectra are outliers.
- Removal of outliers: The identified outlier spectra are removed from the original dataset, hopefully improving the robustness and accuracy of the model.
- Iterating: This entire process can be repeated as needed, each time recalculating the errors for each spectrum and identifying and removing outliers.

The advantage of this method is that it can help to increase the robustness of the PLS models by removing outliers that would otherwise distort the model parameters. It is a relatively simple and intuitive approach that combines the robustness of resampling with the ability to identify and remove problematic data points. This method helps to further reduce the Root Mean Square Error of Prediction (RMSEP) and thus improve the overall performance of the model.

However, as with any method, it should be used judiciously. Removing outliers too aggressively can lead to over-fitting, where the model becomes over-fitted to the “typical” data points and performs poorly on new, unknown data. This method is most useful if you have a large enough dataset so that removing some data points does not significantly reduce the overall size of the dataset.

## 3. Simulator Construction

In order to develop a predictive control algorithm for automated nutrient feeding in a bioreactor, a simulation environment based on a dynamic model was implemented. The latter describes the kinetics of the growth of a CHO cell culture in a fed-batch bioreactor. It is well known that the process parameters (temperature, pH, feeding, ammonia removal, etc.) have a significant impact on cell growth and especially on the quality of the monoclonal antibodies (mAbs) produced [45]. Therefore, the model is important not only for the development of management algorithms, but also for the observation and identification of the key factors (variables and parameters) that have the greatest influence on cell productivity. This is particularly important from the point of view of optimising protein production in a mammalian cell line.

### 3.1. Modelling CHO Cell Culture Kinetics

Chinese Hamster Ovary cells are the most commonly used mammalian hosts for the industrial production of therapeutic proteins, due to their capacity to perform human-like post-translational modifications. The growth kinetics of CHO cells can be studied using a mechanistic model [32]. A mechanistic model is a type of model used to describe biological processes based on underlying physiological mechanisms. These models allow us to interpret, predict and simulate biological phenomena by using mathematical equations to represent the interactions and transformations that occur in a system. In the context of CHO cell growth kinetics, a mechanistic model would include at least the following components. One of the most important mechanisms determining the growth kinetics of CHO cells is cell division. The rate at which cells grow and divide depends on various influencing factors such as the availability of nutrients, the accumulation of waste products and the passage of time. Mathematical models such as the Gompertz model or the logistic growth model are often used to represent these complicated dynamics of cell growth. Another crucial determinant of cell growth is the assimilation and utilisation of nutrients such as glucose and glutamine. The rate at which these nutrients are consumed can have a significant impact on cell growth and is usually modelled using Monod or Michaelis–Menten kinetics, which provides essential insights into cell metabolism and growth patterns. As cells grow and metabolise nutrients, they inevitably generate waste products such as lactate or ammonia. The accumulation of these waste products can have a suppressive effect on cell growth. To quantify this inhibitory effect, mathematical models are used to provide detailed insight into the relationship between the accumulation of waste products and cell proliferation. The loss of cells through mechanisms such as apoptosis, nutrient deprivation or the toxic effect of accumulated by-products is an inevitable aspect of cell culture. Mathematical models are used to express the rate of cell death as a function of various parameters, providing valuable insights into the factors that influence cell viability over time. Finally, the growth kinetics of CHO cells are significantly influenced by external environmental factors such as temperature, pH and osmolality. These factors must be carefully incorporated into the mechanistic model to ensure its relevance and accuracy. These environmental influences represent an additional layer of complexity and require a comprehensive understanding of their effects on cell growth and survival. Each of these components is interconnected and forms a complex network of interactions that determine the growth kinetics of CHO cells. Together, they form a robust mechanistic model that allows the prediction, interpretation and simulation of the behaviour of CHO cells under different conditions. A mechanistic model of CHO cell growth kinetics would typically be a system of differential equations, where each equation represents a particular biological process (such as cell growth, nutrient consumption, production of waste products, etc.). These models can be quite complex and usually require a large amount of experimental data for their parameterisation.

However, despite their complexity, mechanistic models can provide valuable insights into the cell growth process and can be helpful in optimising cell culture conditions for maximum productivity. Many authors [45,46,47,48] who have worked on modelling the kinetics of CHO cell cultures have set up various dynamic models in the form of differential equations based on steady-state analysis. In most cases, these simple models only describe the variation of extracellular metabolite concentrations and the number of live/dead cells during the cell cycle. The models differ in the number of factors considered (number of variables and parameters), which are more or less relevant to describe what actually happens in a mammalian cell line (in a bioreactor). However, in order to have a practical and universally applicable simulator, a model was needed that took all the important variables into account. An example of such a model was also developed by M. Ivarsson [48] in her PhD thesis, as it takes into account the four phases of the cell cycle, temperature, glutamine concentration, number of dead cells, etc., in addition to the number of living cells and the concentrations of glucose, lactate and ammonia. For the development of a predictive controller for automated feeding, only a model prediction of glucose concentration would be required at this stage. However, as glucose concentration variations are also highly dependent on other variables, these should also be considered in the model. As mentioned above, the chosen dynamic model [48] describes four phases of the cell cycle: $G_{0}$, $G_{1}$, *S* and $G_{2}/M$ and the number of cells per phase: $X_{G0}$, $X_{G1}$, $X_{S}$ and $X_{G2/M}$:

$G_{1}$ phase:

(12) $$\frac{d(X_{G1}V)}{dt}=2k_{G2/M-G1}X_{G2/M}V-k_{G1-S}X_{G1}V-k_{G1-G0}X_{G1}V-k_{d}X_{G1}V-F_{OUT}X_{G1}$$

*S* phase:

(13) $$\frac{d(X_{S}V)}{dt}=k_{G1-S}m_{stress}X_{G1}V-k_{S-G2/M}X_{S}V-k_{d}X_{S}V-F_{OUT}X_{S}$$

$G_{2}/M$ phase:

(14) $$\frac{d(X_{G2/M}V)}{dt}=k_{S-G2/M}X_{S}V-k_{G2/M-G1}X_{G2/M}V-k_{d}X_{G2/M}V-F_{OUT}X_{G2/M}$$

$G_{0}$ phase:

(15) $$\frac{d(X_{G0}V)}{dt}=k_{G1-S}\left(1-m_{stress}\right)X_{G1}V+k_{G1-G0}X_{G1}V-k_{d}X_{G0}V-F_{OUT}X_{G0}$$

The equations include transition factors *k*, where, e.g., $k_{G1-S}$ represents the transition from the $G_{1}$ phase to the *S* phase. The transition factors between subpopulations depend mainly on the growth rate, which in turn is determined by the times ($t_{G1}$, $t_{s}$ and $t_{G2/M}$) required for the completion of each cellular phase:

(16) $$\mu =\frac{\ln(2)}{t_{G1}+t_{S}+t_{G2/M}}$$

The transition from the $G_{1}$ to the $G_{0}$ phase is determined by the transition factor $k_{G1-G0}$, which represents the temperature stress. However, the transition to phase $G_{0}$ may also cause metabolic stress $m_{stress}$. The number of viable cells is calculated as the sum of the cells from each phase, where *V* represents the current volume of material in the bioreactor:

(17) $$\frac{d(X_{V}V)}{dt}=\frac{d(X_{G0}V)}{dt}+\frac{d(X_{G1}V)}{dt}+\frac{d(X_{S}V)}{dt}+\frac{d(X_{G2/M}V)}{dt}$$

The volume varies depending on the nutrient dosage ($F_{Glc}$ and $F_{Gln}$) and the potential sampling $F_{OUT}$:

(18) $$\frac{dV}{dt}=F_{Glc}+F_{Gln}-F_{OUT}$$

Glutamine concentration varies according to consumption factor $Q_{G}ln$ and degradation to ammonia $K_{deg}$ and potential dose $F_{Gln}$. Glutamine consumption depends on the cell growth factor, the specific yield $Y_{Gln}$ and the limiting function $f_{upt}$:

(19) $$\frac{d(GlnV)}{dt}=-Q_{Gln}X_{V}V-K_{deg}GlnV+F_{Gln}Gln_{Feed}-F_{OUT}Gln$$

The ammonia concentration depends largely on changes in the glutamine concentration, since the ammonia concentration increases with glutamine consumption (factors $Y_{Amn}$ and $K_{deg}$):

(20) $$\frac{d(A_{mn}V)}{dt}=Q_{Gln}Y_{A_{mn}}X_{V}V+K_{deg}GlnV-F_{OUT}Amn$$

The glucose concentration varies according to the consumption factor $Q_{Glc}$ and the minimum consumption to keep the cells alive $m_{Glc}$, and the amount of glucose added $F_{Glc}$. The consumption factor $Q_{Glc}$ is influenced by temperature and lactate as an inhibitor:

(21) $$\frac{d(\mathrm{GlcV})}{dt}=-Q_{\mathrm{Glc}}X_{V}\left(1-f_{G0}\right)V-m_{\mathrm{Glc}}X_{V}f_{G0}V+F_{\mathrm{Glc}}{\mathrm{Glc}}_{\mathrm{Feed}}-F_{\mathrm{OUT}}\mathrm{Glc}$$

The lactate concentration depends on the glucose consumption ($Q_{Glc}$ and $m_{Glc}$):

(22) $$\frac{d(LacV)}{dt}=Y_{Lac}Q_{Glc}X_{V}\left(1-f_{G0}\right)V-Y_{Lac}m_{Glc}X_{V}f_{G0}V-F_{OUT}Lac$$

The change in monoclonal antibody concentration is determined by factors representing the productivity level ($q_{G1/G0}$, $q_{S}$ and $q_{G2/M}$) per cell phase:

(23) $$\frac{d(mAbV)}{dt}=\mu \left[q_{\frac{G1}{G0}}\left(X_{G1}+X_{G0}\right)+q_{S}X_{S}+q_{\frac{G2}{M}}X_{\frac{G2}{M}}\right]-F_{OUT}mAb$$

## 4. Results

In order to be able to monitor the process in the bioreactor in detail during the entire batch, which usually takes about 14 days, seven PLS models were developed in the Matlab environment. The latter models, which represent soft sensors, allow the monitoring of the most important process variables in CHO cell cultivation. These variables are: Glucose concentration, viable cell concentration (VCC), total cell count (TCC), glutamine, glutamate, lactate and ammonium.

Data from four different batches were available to us for the development of PLS models. Raman spectra were collected every half hour and reference measurements (offline) were performed once or twice a day with Cedex Analyzer. Thus, the first step was to find the pairs of spectra and reference measurements that matched best in terms of acquisition time. The Raman measurement takes about half an hour to obtain a good signal-to-noise ratio and to remove fluorescence interference.

As described in Section 2.1, two key initial steps in the development of PLS models are the preprocessing of the Raman spectra with the Savitzky–Golay filter and the normalisation with the Standard Normal Variate method (see Figure 2 and Figure 3). Savitzky–Golay low-pass filtering was performed for all independent variables (Raman shift (cm${}^{-1}$)) of each spectrum, with a quadratic function chosen for smoothing with the Savitzky–Golay filter and the window length (smoothing) set to 15 samples. In addition, a normalisation or Standard Normal Variate function is applied to the independent variables for all spectra, resulting in spectra with a mean of zero and a standard deviation of one.

As described in Section 2.2 and illustrated in Figure 2, careful consideration is also required in the selection of the parameter that determines the number of latent variables. For each PLS model, the optimal number of latent variables is determined based on cross-validation, aiming for the smallest RMSECV error. In general, it is preferred to keep the number of latent variables as small as possible.

Characteristic independent variables of the spectrum (i.e., energy shifts) at which a spike occurs can be extracted from the literature for individual observed variables. Taking these characteristic energy shifts into account when calculating PLS models is therefore considered useful as it further weighs the individual independent variables of the spectrum and improves the model in this way. If these characteristic energy shifts are not known, various methods are available to identify the more important independent variables and take them into account to a greater extent.

The Variable Importance in the Projection method, described in Section 2.2.2, was tested first. However, the prediction results were not improved by this simple method; thus, alternative approaches to selecting key variables were investigated. Attempts to select "key" intervals or several independent variables of the spectrum together also did not lead to better results.

**Figure 7:**
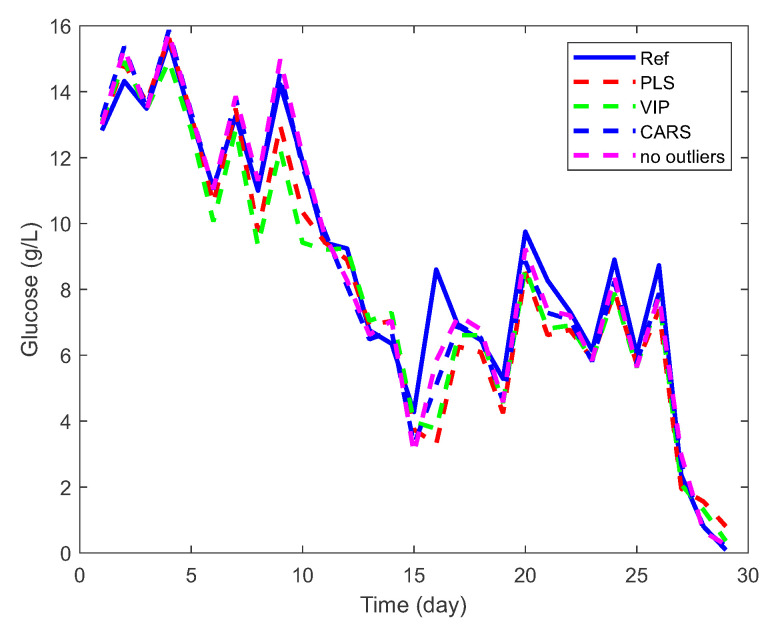
Validation of the PLS glucose model using VIP, CARS and outlier removal methods.

It turned out that the Competitive Adaptive Reweighted Sampling method, which is also discussed in Section 2.2.2, gave the best results for selecting key variables when building PLS models. As can be observed in Figure 5, the method CARS identifies fewer key variables than the method VIP. Nevertheless, the validation results of the PLS model (using glucose concentration as an example) were better when the method CARS was used, as evidenced by the smaller Root Mean Square Error (see Figure 7 and Table 1).

The reference values in Figure 7 represent offline measurements performed with the Cedex Analyzer. In some cases of glucose measurement, the VIP method even leads to worse results than not using a method, as shown in Table 1 (see RMSE).

**Figure 8:**
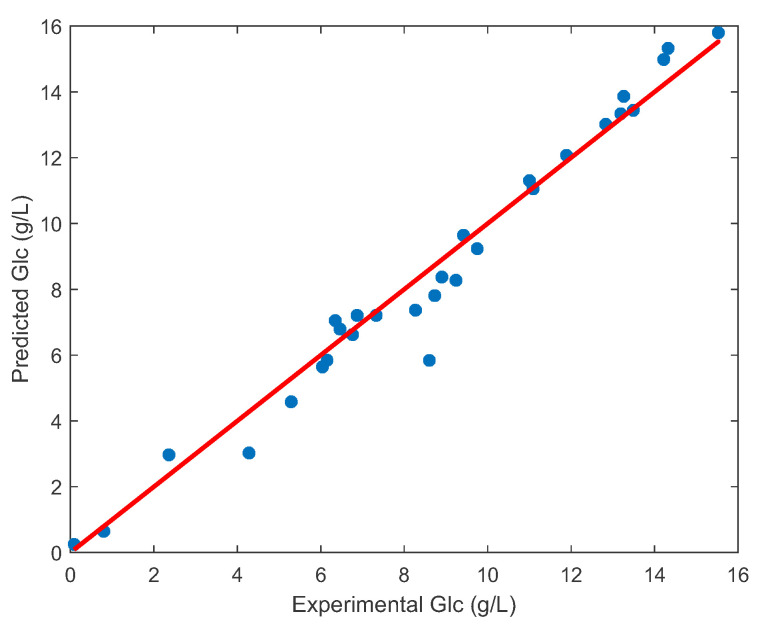
Validation of the PLS glucose model: comparison of experimental and predicted values using CARS and outlier removal methods.

Assuming that Cedex’s offline measurements are reliable, the training set was examined for spectra representing outliers that could affect the parameters of the PLS model during the learning phase and consequently affect the prediction accuracy. Applying the Monte Carlo sampling method and calculating the mean error and standard deviation for each PLS model led to the identification of spectra within the dataset that represent outliers, as shown in Figure 6 and discussed in Section 2.2.3. This process allowed a further increase in the accuracy and robustness of the PLS models, as can be observed in Figure 7 and Table 1. In this case, the coefficient of determination for the PLS model for glucose is $R^{2}=0.97$, which means that the PLS model has been further improved compared to the method CARS (where $R^{2}=0.96$). An accurate prediction of glucose concentration can also be observed in Figure 8, which shows a comparison of experimental and predicted values using CARS and methods to remove outliers. Ideally, all points should lie on a straight line.

Table 2 shows the RMSE and coefficient of determination ($R^{2}$) for the following constructed PLS models in addition to the glucose PLS model: VCC, TCC, glutamine, glutamate, lactate and ammonium. The results demonstrate that all PLS models developed provide an accurate prediction of the main process variables ($R^{2}>0.8$), and only the PLS model for glutamine has a slightly worse prediction ($R^{2}=0.33$). The reason for this lies in the following fact. In Raman spectroscopy, glutamine and glutamate are related because they have a similar molecular structure and similar active Raman vibrational modes that produce similar spectral features. Glutamine and glutamate are structurally similar amino acids, both containing a carboxyl group (-COOH) and an amine group (-NH2). The main structural difference between them is that glutamate has an additional carboxyl group, while glutamine has an amide group (-CONH2) instead. It is important to note that while Raman spectroscopy is a powerful technique for identifying molecules, its resolution is often insufficient to distinguish between similar molecules in a mixture. In such cases, additional techniques, such as chromatographic separation or more sophisticated spectral analysis methods, are required.

Table 3 shows the best RMSE results for PLS models according to the existing literature [11,37]. A comparison with the data in Table 1 and Table 2 shows that our method for building PLS models excels at accurately predicting key variables from Raman spectra. This comparison essentially underlines the effectiveness of our approach. It is particularly noteworthy that our PLS models have an RMSE that is on average three times smaller than the RMSE published in recent research [11,37].

**Figure 9:**
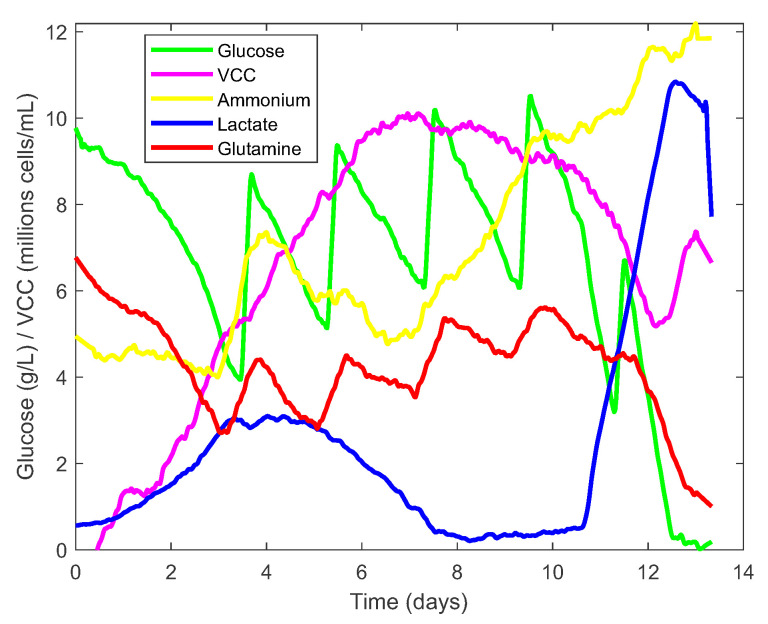
Signal reconstruction of key process variables via PLS models.

The learning process for the PLS models depended on a single offline measurement (Cedex) of each variable (e.g., glucose) per day. Therefore, only the Raman spectroscopy spectra that matched the offline measurements in time could be used. However, once the PLS models were built, all spectra collected every half hour could be used, giving an informative representation of the time course of each variable (see Figure 9). These data are then used in the optimisation to determine the parameters of the dynamic model for the CHO cell kinetics, as described in the Section 3.1. Careful examination of the time series signal for glucose and glutamine concentrations in Figure 9 reveals a sawtooth pattern due to the daily manual dosing of nutrients. This pattern is not conducive to the optimal growth of the CHO cells.

**Figure 10:**
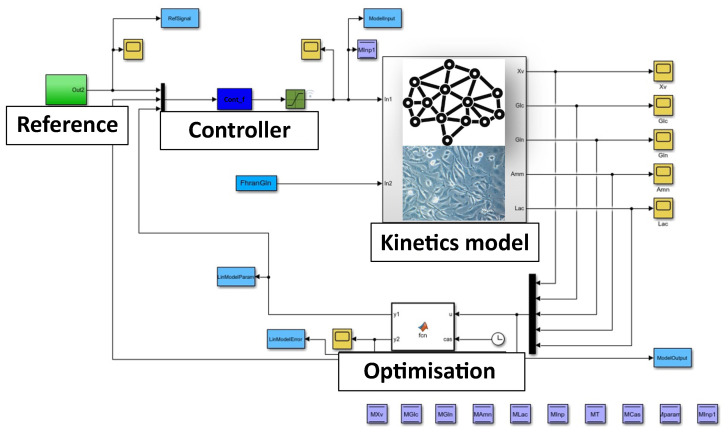
Implementation of a simulator within the Simulink environment based on the CHO cell kinetics model.

The problem can be solved by implementing an automated feeding system that continuously doses the nutrients according to a predefined reference signal. However, such a system requires not only the application of the previously developed soft sensors (PLS models), but also a simulation environment. In this environment, a control algorithm can be developed and different scenarios such as different feeding regimes, the removal of inhibitors and the observation of important process variables can be investigated. The heart of the simulator, represented by the Simulink schema in Figure 10, is a dynamic model of CHO cell kinetics, which is explained in the Section 3.1. Figure 10 also shows the controller and optimisation blocks, the details of which will be explained in more detail in forthcoming scientific publications.

Based on known process parameters (temperature and pH) and time series signals of the main process variables (VCC, glucose, glutamine, etc.), it is possible to perform the optimisation of the parameters of the dynamic model of CHO cell kinetics (presented in the Section 3.1). This optimisation aims at aligning the model results as much as possible with the measurements of previous batches.

For the parameter optimisation, the particle swarm optimisation (PSO) method was used, which makes it possible to find the global minimum of the chosen criterion function while optimising a large number of parameters. In this case, the criterion function was given as RMSE, with the final values presented in the Table 4.

**Figure 11:**
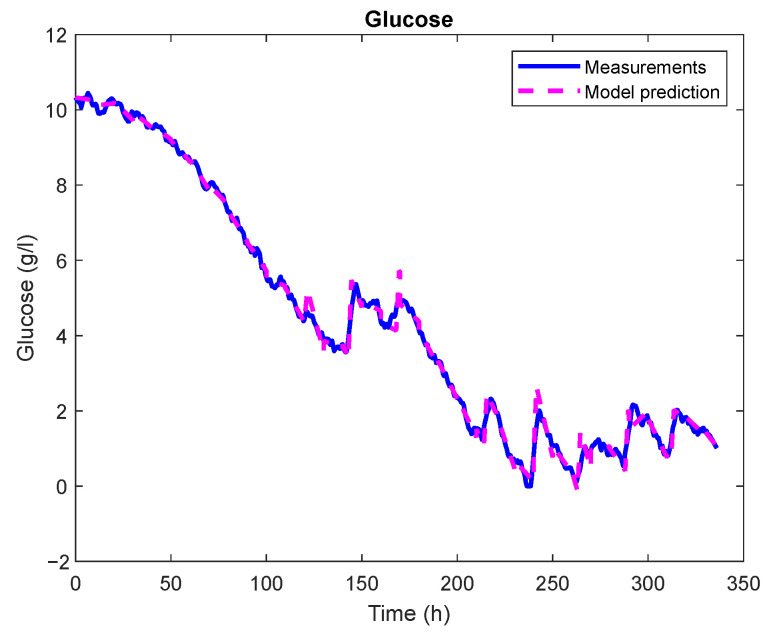
Validation of the CHO cell kinetics model in the case of glucose concentration prediction for the entire batch run.

A comparison of glucose concentration measurements from one of the batches with a glucose concentration prediction derived from a mechanistic model of CHO cell kinetics is shown in Figure 11. The results of the agreement were excellent in this case, with an RMSE of 0.18 g/L and $R^{2}=0.99$.

**Figure 12:**
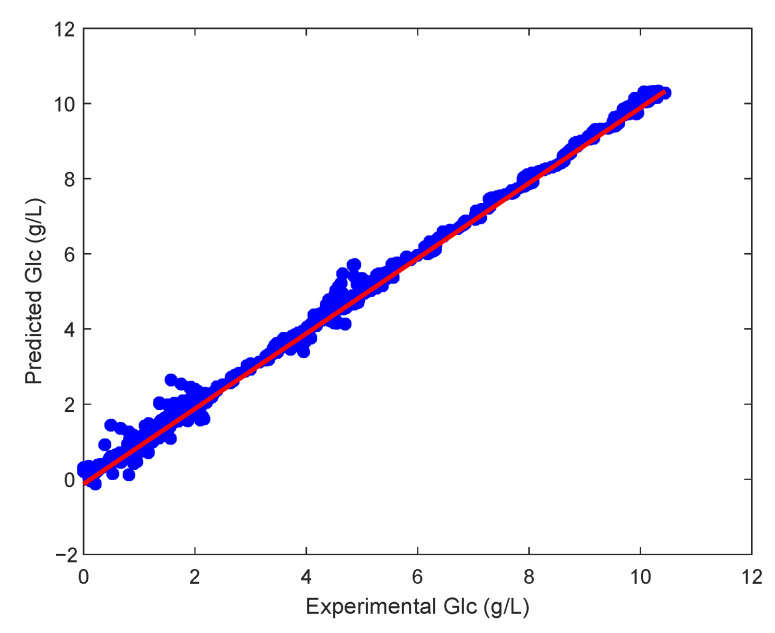
Validation of the CHO cell kinetics model: comparison of experimental and predicted glucose concentrations.

Furthermore, Figure 12 shows the remarkable matching between the measurements and the predicted values; ideally, all points should lie on a straight line. However, it is important to note that the available data were limited to only four batches. If a larger number of batches are included in the optimisation process, a slight deviation between the individual batches and the process variables is to be expected. In the future, it would be beneficial to combine the data from the individual batches based on the criterion of mutual similarity and then determine the model parameters for the individual clusters.

The predictions for the other process variables, as shown in Table 4, prove satisfactory when the CHO cell kinetics model is used. Only in the case of glutamine concentration does a somewhat larger error occur, which has already been pointed out. The reason for this is that when the PLS model predicts the time series signal for glutamine with less accuracy, the variance of the “measurements” (derived from the soft sensor) increases. Consequently, the time series signal of glutamine is predicted with lower accuracy by the mechanistic model.

## 5. Discussion

In developing models that allow the use of soft sensors to monitor key process variables (VCC, TCC, glucose, glutamine, glutamate, lactate and ammonium) in the bioreactor, it was found that using the PLS method alone did not provide the required accuracy and robustness of the models. In particular, with a limited data set (a few batches), the model can be overfitted, leading to a sharp drop in predictive performance compared to what the validation with limited data promises.

Since in our work only about 100 spectra with reference measurements were available during the learning phase and Raman spectra contain more than 3000 components, the phase of selecting key variables became crucial for model construction. By using the CARS method, better handling of collinearity between variables was observed, as well as better performance on small data sets and higher robustness compared to the VIP method. As a result, the RMSE was reduced by up to 30%.

It was found that the VIP method further impaired the predictive ability of the models in certain cases, indicating an overfitting problem, as the number of key variables selected was significantly larger than required by the CARS method. The VIP method also had stability problems, as the results may have become unstable with small samples. Minor variations in the data can lead to significant shifts in the scores, making it difficult to extrapolate the results to other data sets. When calculating the VIP scores based on the weighted sum of squares of the PLS loadings, high variability was found in small data sets.

In Raman spectroscopy, it is important to understand that outlier spectra can occur, influenced by various factors. For example, if the sample in the bioreactor is not evenly mixed, this can lead to deviations in the spectra obtained. Raman spectroscopy derives its readings from the average properties of the area illuminated by the laser. Therefore, a lack of homogeneity in the sample can lead to inconsistent measurements.

Moreover, the components of the sample can play an important role. If components fluoresce under the laser light of the Raman spectrometer, the resulting fluorescence could overshadow the Raman signal and distort the spectra. Additionally, bubbles or particles in the bioreactor can cause scattering or absorption of the laser light, resulting in unpredictable spectra.

Given these potential sources of error, it is important to carefully identify and remove outlier spectra during the modelling phase, as described in Section 2.2.3. This step reduced the root mean square error (RMSE) by 10% (in addition to 30% reduction with the method CARS).

The efficient growth and production of desired products by CHO cells requires specific, strictly controlled conditions in the bioreactor. These conditions include the regulation of pH and temperature, which affect cell metabolic rate, protein folding and expression levels. Equally important is the careful control of nutrient content, especially glucose and glutamine, according to a predetermined profile for the duration of the batch.

Another critical factor is the control of inhibitor concentrations. Metabolic by-products such as ammonia and lactate can potentially inhibit cell growth and protein production if they reach high concentrations. Since glucose is the primary source of energy, its concentration directly affects cell metabolism. Too little glucose can starve cells and inhibit growth, while too much glucose can cause osmotic stress or trigger overproduction of waste products such as lactate.

Given these complexities, the use of an automated bioreactor control system is essential for CHO cell cultivation. Such a system offers several advantages, including maintaining consistent conditions, real-time monitoring, reducing human error and improving efficiency and scalability. Given the significant costs associated with realistic bioreactor experiments, the development of a simulation environment is essential. This environment enables the creation of control algorithms and the evaluation of the effects of different parameters on cell growth and productivity.

The main reason for the lack of advanced automated control techniques in cell culture bioprocesses and bioreactor operations is that these techniques require robust and reliable measurement methods that are available on site. Concentrations of nutrients and metabolites, cell densities and viability are not measured and are uncontrolled or are only controlled manually with long sampling times (12–24 h, as shown in Figure 9). As a result, possible process disturbances may only be detected after long delays, making it difficult to take corrective action and increasing the risk of batch losses.

For the development of an advanced simulation environment, the choice of a CHO kinetic model is also crucial. The chosen model should represent the complex kinetics of CHO cells in sufficient detail. Simpler models based on the Monod equation, for example, are often inadequate in this respect. More complex models, however, pose the challenge of determining numerous parameters that can only be accurately determined with a suitable optimisation method and sufficiently heterogeneous data. In our study, the parameters of a dynamic model of CHO cell kinetics were successfully determined using the PSO method.

To enable the development of a predictive control algorithm, the complex kinetics model will be simplified and linearised, and online adjustment of the (adaptive) model parameters will be facilitated. This adjustment is made possible by an optimisation method that uses the measurements of the current batch to facilitate the online parameterisation.

Future efforts include the development of a model predictive control algorithm based on the simplified model of CHO cell kinetics. Subsequently, the monitoring and control system will be integrated into a real bioreactor. Finally, a practical test of the implemented system will be carried out.

## 6. Conclusions

This study demonstrates the significant advances in fully automated feeding of CHO cells achieved through the development of advanced models, soft sensors and a novel simulation environment. The research has required a thorough understanding of various chemometric methods and demonstrated their context-specific application in combination with Raman spectroscopy. It has demonstrated the effectiveness of CARS-PLS and an outlier removal method in overcoming difficult challenges such as high dimensionality, multicollinearity and outlier detection. The models created are versatile and scalable and can be applied to a wide range of products, media and cell lines based on CHO host cells. They can be conveniently scaled up for use in large pilot studies and extensive manufacturing processes. However, the success of these methods depends not only on the right choice of techniques, but also crucially on the quality of the input data. Therefore, the preprocessing of the data to remove interfering signals is of the utmost importance. Raman spectra have no inherent value, but when integrated with the appropriate models, they allow for the creation of a sophisticated measurement system. This system, which consists of soft sensors, is used for real-time monitoring and control of important process variables. The measurements reconstructed with these soft sensors play a crucial role in the design of the simulation environment, which significantly speeds up and cheapens the development of control algorithms and thus the automated nutrient dosing system. In essence, this study provides essential insights into the pragmatic application of Raman spectroscopy and innovative methods that form a solid foundation for further research and development in the field of automated cell feeding.

## Figures and Tables

**Table 1:** Root Mean Square Error of glucose concentration prediction and the coefficient of determination ($R^{2}$).

	RMSE (g/L)	$R^{2}$
PLS	1.25	0.92
VIP	1.26	0.92
CARS	0.84	0.96
No outliers	0.75	0.97

**Table 2:** Root Mean Square Error and the coefficient of determination ($R^{2}$) for all other constructed PLS models.

	RMSE	$R^{2}$
VCC	0.86 ($10^{6}$ cells/mL)	0.93
TCC	1.06 ($10^{6}$ cells/mL)	0.91
Glutamine	1.60 (g/L)	0.33
Glutamate	0.26 (g/L)	0.95
Lactate	0.10 (g/L)	0.99
Ammonium	1.16 (mmol/L)	0.83

**Table 3:** The best RMSE results for PLS models found in the literature [11,37].

	RMSE
VCC	4.87 ($10^{6}$ cells/mL)
TCC	3.68 ($10^{6}$ cells/mL)
Glucose	1.13 (g/L)
Glutamate	1.18 (g/L)
Lactate	0.19 (g/L)
Ammonium	1.21 (mmol/L)

**Table 4:** Root Mean Square Error and the coefficient of determination ($R^{2}$) in the case of predicting all important process variables using the CHO cell kinetics model.

	RMSE	$R^{2}$
VCC	0.15 ($10^{6}$ cells/mL)	0.99
Glucose	0.18 (g/L)	0.99
Glutamine	0.20 (g/L)	0.98
Lactate	0.14 (g/L)	0.99
Ammonium	0.10 (mmol/L)	0.99

## Data Availability

The data presented in this study are available on request from the corresponding author. The data is not publicly available due to trade secrets.
